# An economic evaluation of adaptive e-learning devices to promote weight loss via dietary change for people with obesity

**DOI:** 10.1186/1472-6963-12-190

**Published:** 2012-07-07

**Authors:** Alec Miners, Jody Harris, Lambert Felix, Elizabeth Murray, Susan Michie, Phil Edwards

**Affiliations:** 1 Faculty of Public Health and Policy, London School of Hygiene and Tropical Medicine, Keppel Street, London, WC1E 7HT, UK; 2 Faculty of Epidemiology and Population Health, London School of Hygiene and Tropical Medicine, Keppel Street, London, WC1E 7HT, UK; 3 Research Department of Primary Care and Population Health, University College London, Upper Floor 3, Royal Free Hospital, Rowland Hill Street, London, NW3 2PF, UK; 4 Research Department of Clinical, Educational & Health Psychology, University College London, 1-19 Torrington Place, London, WC1E 7HB, UK

**Keywords:** Economic evaluation, e-learning, cost-utility analysis, obesity and weight management.

## Abstract

**Background:**

The prevalence of obesity is over 25 % in many developed countries. Obesity is strongly associated with an increased risk of fatal and chronic conditions such as cardiovascular disease and type 2 diabetes. Therefore it has become a major public health concern for many economies. E-learning devices are a relatively novel approach to promoting dietary change. The new generation of devices are ‘adaptive’ and use interactive electronic media to facilitate teaching and learning. E-Learning has grown out of recent developments in information and communication technology, such as the Internet, interactive computer programmes, interactive television and mobile phones. The aim of this study is to assess the cost-effectiveness of e-learning devices as a method of promoting weight loss via dietary change.

**Methods:**

An economic evaluation was performed using decision modelling techniques. Outcomes were expressed in terms of Quality-Adjusted Life-Years (QALYs) and costs were estimated from a health services perspective. All parameter estimates were derived from the literature. A systematic review was undertaken to derive the estimate of relative treatment effect.

**Results:**

The base case results from the e-Learning Economic Evaluation Model (e-LEEM) suggested that the incremental cost-effectiveness ratio was approximately £102,000 per Quality-Adjusted Life-Year (QALY) compared to conventional care. This finding was robust to most alternative assumptions, except a much lower fixed cost of providing e-learning devices. Expected value of perfect information (EVPI) analysis showed that while the individual level EVPI was arguably negligible, the population level value was between £37 M and £170 M at a willingness to pay between £20,000 to £30,000 per additional QALY.

**Conclusion:**

The current economic evidence base suggests that e-learning devices for managing the weight of obese individuals are unlikely to be cost-effective unless their fixed costs are much lower than estimated or future devices prove to be much more effective.

## Background

According to recent Organisation for Economic Co-operation and Development (OCED) data, the prevalence of obesity has increased to almost 25 % in countries such as England, Canada, Ireland and Australia and to over 30 % in Mexico and the US [1]. Obesity is associated with a higher risk of many diseases, including cardiovascular disease and type 2 diabetes [2-5]. It is also associated with significant costs to the health care- and other sectors. Unsurprisingly therefore, designing and delivering effective weight management interventions has become a priority in many countries and a focus for many public health programmes [5,6].

Interventions to change dietary behaviour are an important method of tackling obesity [7-9]. Specifically, interventions designed to modify or replace diets high in saturated fats and sodium with those containing more fruit, vegetables and lower saturated fats. Indeed the WHO reports that the consumption of up to 600 g per day of fruit and vegetables could reduce the total worldwide burden of disease by 1.8 %, and reduce the burden of ischaemic heart disease and ischaemic stroke by 31 % and 19 % respectively [10].

A new and evolving area in the promotion of dietary behavioural change is ‘e-Learning’, the use of interactive electronic media to facilitate teaching and learning on a range of issues including health. E-Learning devices (e-LDs) have grown out of recent developments in information and communication technology, such as the Internet, interactive computer programmes, interactive television, and mobile phones [11-15]. So-called ‘second and third generation’ e-Learning interventions use ‘adaptive’ interactive technology delivered on computers and portable devices, such as mobile phones to produce iterative, interactive and more immediate feedback [16]. Moreover, they are rapidly becoming more accessible to the general population (e.g., an estimated 70 % of the population in the UK has access to the internet and this percentage is likely to continue to grow [17]). The high level of accessibility, combined with emerging advances in computer processing power, data transmission and data storage, makes interactive e-LDs a potentially powerful and cost-effective medium for improving dietary behaviour and ultimately health [18-20]. However, while the general conclusion from three systematic reviews is that they show some promise in terms of dietary behaviour change [16,21,22], and a fourth suggests some encouragement in terms of weight loss [23], a number of important uncertainties remain. For example, the most recent review included studies published up to 2008 [16], they were restrictive in terms of patient entry criteria and, importantly, none assessed cost-effectiveness. The latter point is the focus of this study.

## Methods

### Decision problem and model basics

An economic decision model was built, referred to as the e-Learning Economic Evaluation Model (e-LEEM), to assess the cost-effective of the e-LDs. The model consists of a cost-utility analysis (CUA), with health outcomes expressed as quality-adjusted life-years (QALYs). Costs were assessed from a UK health services perspective, and expressed in 2009 prices. In all scenarios, the model was run until all patients died, implying a lifetime horizon for the analysis. All future costs and QALYs were discounted at 3.5 % per annum.

Defining how an e-LD is used, and therefore its associated costs and outcomes, is difficult because they are idiosyncratic in terms of design, platform base, not all are commercially available and they and their use are often poorly described in the clinical trials. Thus, for the purpose of the economic evaluation a single hypothetical/generic package has been defined broadly reflecting the design and cost of an internet-based intervention evaluated in McConnon et al. [24] as it was a well described contemporary UK-based randomised controlled trial.

There are a number of possible comparator interventions for e-LDs, indeed weight management interventions are rarely used in isolation as results from the systematic review suggest [25]. For this reason, and to be practical, a generic ‘conventional care’ arm was specified as the comparator intervention, implying an intervention that could contain a number of interventions such as generic dietary information and/or exercise but excluding interventions based on e-LDs or pharmacological treatment. The latter was included as a third treatment option in a sensitivity analysis in an attempt to put the results into a broader context. Note however, that the estimate of relative clinical effect was not based on formal indirect or mixed treatment comparisons.

A number of different modelling approaches were considered. The decision was made to use a discrete event simulation (DES) so that the likelihood of future clinical events and associated costs could be directly linked to individuals’ current health. Simulations were based on 1,000 outer (second order or probabilistic) simulations and 1,000 inner (first-order or micro) simulations. EVPI calculations were also undertaken based on a further 1,000 third-order simulations. The model was built in TreeAge Pro 2009 [26].

### The decision problem

**Figure 1:**
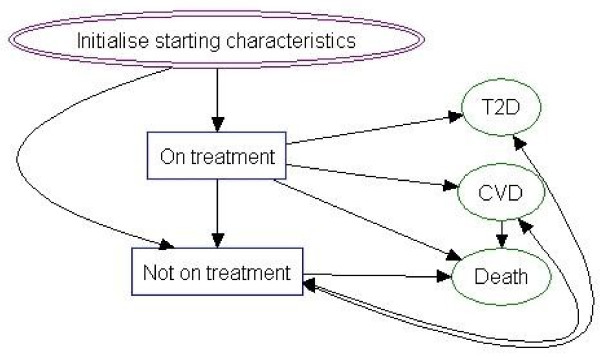
Model schematic

Although e-LDs can be used to prevent obesity and as a means of managing individuals who are already considered obese, it was understood that they are most likely to be used in the latter scenario. Exact patient starting characteristics were changed according to the model run (Table 1) so that issues of patient heterogeneity could be assessed, but in all instances, individuals had body-mass indexes (BMIs) of at least 30 kg/m^2^ with the aim of reducing weight or modifying further increases. Unless otherwise stated, all individuals were assumed to be aged 50 years. All individuals were assumed to receive treatment with either an e-LD or CC for 12 months, or until they developed a disease (type 2 diabetes or cardiovascular disease), died or dropped-out from treatment, whichever event occurred first (Figure 1). A ‘minimising the time to the next event’ approach was used to select the sequence of subsequent (competing) events, given relevant life-tables and risk equations. BMI was chosen as the main model ‘driver’. That is weight change, should it occur, was transformed into a BMI, which in turn affected the time to future events. This meant that, all else remaining equal, the time to developing cardiovascular disease and type 2 diabetes was shorter in individuals with relatively high BMIs compared with individuals with lower BMIs. The natural history of disease in terms of BMI change was also modelled, meaning that as people aged, mean BMI increased, as did the likelihood of disease and death due to obesity. Additionally, the risk equations were linked so, for example, individuals with type 2 diabetes were at increased risk of developing cardiovascular disease compared to those without it.

**Table 1:** Starting characteristics for the base case analyses

**Scenario**	**A**	**B**	**C**	**D**	**E**	**F**	**G**	**H**
*Characteristic*								
BMI	30	30	33	30	33	35	35	30
Sex	Male	Male	Male	Female	Male	Female	Male	Female
Smoker	No	Yes	No	No	Yes	Yes	Yes	Yes
T2D	No	No	No	No	No	No	Yes	No

### Estimating time to events and natural history

The time to death was calculated using a life-table approach, as outlined by Barton et al. [27][28]. In addition to this, 33 % [29] of patients who developed cardiovascular disease (CVD) were assumed to die immediately once the event had occurred. All deaths from T2D were considered to be attributable to cardiac problems, thus they were not independently modelled. These approaches were used in the base case to avoid the possibility of double counting occurrences of death, thus it is arguably conservative. The QRISK2 [30] algorithm was used to predict the risk of CVD. Two T2D risk equations were identified [31][32]. While an arbitrary decision was made to use the Stern equation, the Lindström model was used in a sensitivity analysis.

While receiving treatment with either an e-LD or CC, the baseline annual bodyweight was assumed to change in line with results from a recently reported UK-based RCT [kg ~ N −1.9, 0.63] [24]. Following treatment cessation, the natural history of BMI change was modelled using evidence cited in the 2005 NICE Obesity Guideline [33]. Specifically Fine et al., [34] state that the mean increase in weight over 4 years is 1 kg per year. The Guideline also states that this finding is consistent with the findings of Heitmann et al. [35] who performed a retrospective semi-longitudinal study to determine the pattern of weight changes over 11 years in a Danish population that became overweight in adulthood. Thus, all patients were assumed to put on an average of 1 kg per year independently of initial BMI levels.

Weight gain was converted into an increased BMI by assuming men were on average 1.75 ms tall whereas women were 1.62 ms tall [36]. This is equivalent to a 0.33 and a 0.38 unit increase in BMI respectively, per 1 kg increase in weight [BMI = weight (kg)/height^2^ (m)] [37]. This meant, for example, that it would take an average of 0.31 years (0.1/0.33) for a man’s BMI to increase by 0.1 kg/m^2^.

High attrition rates are a defining feature of weight loss/preventing weight gain interventions. Therefore the probability that individuals stopped treatment before 12 months, for reasons other than developing disease or death, was included. The Turnin 2001 RCT was used to estimate the base case probability of drop out, although this value was altered in a sensitivity analysis. The results showed that 179/557 participants were lost to follow-up over the 12 month period. Differences in attrition rates between the e-LD and CC treatment options were judged to be negligible and were not included in the model [25].

### Estimating relative intervention effect

**Figure 2:**
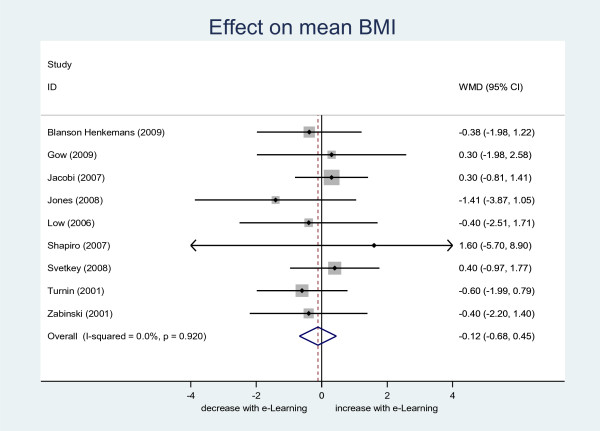
**Forest plot of treatment effect on mean BMI (kg/m**^**2**^

Relative treatment effects were estimated using results from a systematic review and meta-analysis [25]. In brief, interventions were included in the review if they contained RCTs of interactive computer software programmes that tailored output according to user input, in adolescents or adults, did not include any human interaction (eg. motivational telephone calls) where the focus was on promoting dietary change. In all, 43 eligible RCTs were identified, of which 9 (33 %) reported mean BMI at the study end with standard deviations [25]. Random effects meta-analysis suggested a non-statistically significant (p = 0.69) weighted mean difference in favour of the e-LDs [N ~ −0.12, 0.29] (Figure 2). There was evidence to suggest estimates of effect did not differ according to whether follow-up was earlier (within 3 months) or later (p = 0.59), if they included participants who were overweight (p = 0.58), whether the studies aimed to maintain or reduce BMI (p = 0.91) or whether interventions included a physical activity component (p = 0.91).

The cost-effectiveness of orlistat was also estimated in a single sensitivity analysis. Orlistat’s mean annual effect was estimated to be a loss of 3.46 kg, as reported by a review of three RCTs [38].

### Costs

Costs were broadly divided into two types: those associated with specific events, and those relating to the initial 12 months of treatment (Table 1).

All costs were inflated to 2009 prices using the Hospital and Community Health Services Index [39]. The costs of CVD were taken from Warren et al., [40] and were reported as one-off costs for fatal, non-fatal events and an annual cost for survivors. No other costs associated specifically with CVD were included. The annual cost of diagnosed T2D was taken from Ara [41]. The cost included 2 general practitioner visits per year, a specialist nurse visit, drug treatment for high blood pressure, statin therapy and treatment with metformin. The annual costs of CC and those associated with e-learning devices were taken from a UK-based RCT of web-based support package [24]. The costs for both interventions included resources such as drug costs and health care visits and slimming clubs. The main difference between the two was that the internet-based support package included an additional fixed cost per patient of £854 per annum for the actual web-based support (meaning this cost was applied per patient irrespective of how long their treatment lasted in the base case). The yearly cost of orlistat treatment was assumed to be £415 (based on 120 mg treatment 3 times per day, at a unit price per pill of £0.38 [£32.27/84]), plus the cost of 5 GP visits (£35 per visit).

### Utilities

All utility values were estimated from a single large UK study that assessed the relationship between BMI, other health-related issues and EQ-5D utility scores (Table 2) [42]. A maximum of two long-standing illnesses were permitted, representing the possibility of developing CVD and T2D.

**Table 2:** Description of costs

**Description**	**Mean Value**	**Distribution^**	**Source**
**CVD fatal event**	£3,058	Gamma (9, 0.00295)	Warren* [40]
**CVD non-fatal event**	£3,648	Gamma (9, 0.002469)	Warren* [40]
**Annual cost of non-fatal CVD event**	£876	Gamma (9, 0.010309)	Warren* [40]
**Annual cost of T2D**	£724	Gamma (9, 0.0125)	Ara [41], NICE Guideline* [33]
**Annual cost attributable to orlistat**	£715	N/A	BNF [43], Foxcroft [38]
**Annual cost of e-leaning device**	£140 (SE £234)	Gamma (0.3549, 0.002535)	McConnon^$^[24]
**Fixed cost of e-learning device**	£854	N/A	McConnon^$^[24]
**Annual cost of conventional care**	£226 (SE £329)	Gamma (0.479, 0.002088)	McConnon^$^[24]

*Indicates that mean values were derived from this source but measures of variance were not reported. In these instances, standard errors were based on one third of the mean value.

^$^An extended report relating to the published economic was supplied by the authors.

^parameterised as required in TreeAge Pro 2009, Gamma ~ (α, λ).

## Results

**Figure 3:**
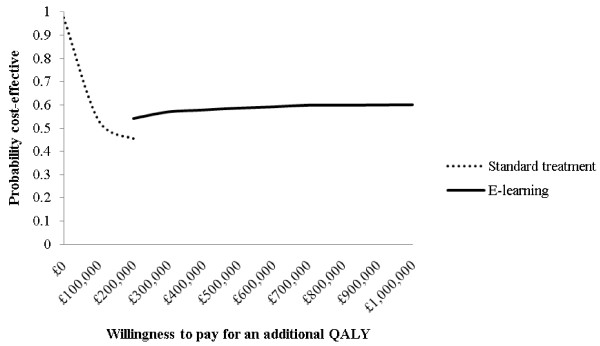
Cost-effectiveness acceptability frontier (CEAF) relating to scenario A

The base case results are shown in Table 3 for a number of different patient starting characteristics. Although the absolute costs and QALYs vary across the scenarios, in each instance the incremental health gains were small, as indicated by the fact very few additional cases of T2D or CVD were averted. The lowest reported ICER was approximately £102,000 per additional QALY (scenario A). Scenarios containing women were associated with lower QALYs compared with men (e.g. scenario D compared with A) because lower rates and time spent with CVD were more than offset by higher rates and time spent with T2D. The cost-effectiveness acceptability frontier for scenario A (Figure 3) shows that up to about £200,000 per additional QALY, CC is the preferred option, but even after this point, there is a high degree of uncertainty around e-LD being the most cost-effective option.

**Table 3:** **Base case results (see Table**4 for patient starting characteristics

**Scenario**	**Intervention**	**Cost (£)**	**QALYs**	**ICER (£)**
A	CC	4,884	12.527	-
	e-LD	5,646	12.534	102,112
B	CC	5,364	12.093	-
	e-LD	6,129	12.100	121,856
C	CC	5,340	12.196	-
	e-LD	6,088	12.200	184,962
D	CC	4,035	11.703	-
	e-LD	4,732	11.708	125,891
E	CC	5,810	11.838	-
	e-LD	6,566	11.844	150,865
F	CC	5,201	11.209	-
	e-LD	5,902	11.214	151,142
G	CC	15,014	10.910	-
	e-LD	15,789	10.911	232,911
H	CC	4,469	11.500	-
	e-LD	5,186	11.506	112,628

CC, conventional care; e-LD, e-learning device; QALYs, quality-adjusted life-years; ICER, incremental cost-effectiveness ratio.

**Table 4:** **Relationship between independent variables and EQ-5D utility scores, Macran et al.**[42]

**BMI group (kg/m**^**2**^**)**	**Coefficient**	**Age group (years)**	**Coefficient**	No. of LSIs*	**Coefficient**
**<21**	−0.02	18-24	0	0	0
**21-25**	0	25-34	0.0005	1	−0.115
**26-30**	−0.02	35-44	−0.01	2	−0.196
**31-39**	−0.04	45-54	−0.02		
**>39**	−0.06	55-64	−0.04		
		65-74	−0.04		
		>75	−0.08		

*LSIs, Long standing illnesses.

### Sensitivity analysis

A number of one-way sensitivity analyses were performed. Arguably the most important in terms of large changes to the ICER were the fixed costs associated with the e-learning devices, the relative treatment effect, the rate at which health outcomes were discounted and the duration of treatment effect (Table 5).

**Table 5:** Sensitivity analysis on scenario A patient characteristics

**Parameter**	**CC**		**e-LD**		
	Costs (£)	QALYs	Costs (£)	QALYs	ICER (£)
**Doubling the time to a 0.1 BMI increase after treatment stops**	4,545	12.972	5,302	12.978	122,125
**£0 initial cost for e-learning devices**	4,903	12.475	4,845	12.483	Dom
**Lindstrom T2D risk equation**[32]	4,248	12.812	5,608	12.818	124,813
**Starting age of 30 years**	5,065	17.976	5,820	17.986	74,151
**Starting age of 60 years**	4,577	9.749	5,347	9.754	118,741
**Using estimate of relative treatment effect from Turnin (N ~ −0.6, 0.71)**[44]	4,999	12.802	5,704	12.837	20,053
**Doubling all T2D related costs**	6,819	12.416	7,561	12.435	83,306
**Doubling all CVD related costs**	7,704	12.507	8,458	12.513	100,480
**Doubling the cost of CC**	5,115	12.482	5,706	12.488	86,323
**0 % discount rate for health benefits**	4,943	19.325	5,699	19.338	58,869
**Halving the attrition rate for both treatments**	4,871	12.799	5,662	17.897	84,483
**Treatment for 24 months**	5,600	12.806	5,719	12.817	64,487

Dom, e-learning is ‘dominant’; *59 % probability e-LD is cost-effective at £30,000 per additional QALY; CC, conventional care; e-LDs; e-learning devices.

When orlistat was included as a comparator, it dominated e-LD. Against CC, orlistat cost almost £6,000 per additional QALY.

### Expected value of perfect information (EVPI) analysis

**Figure 4:**
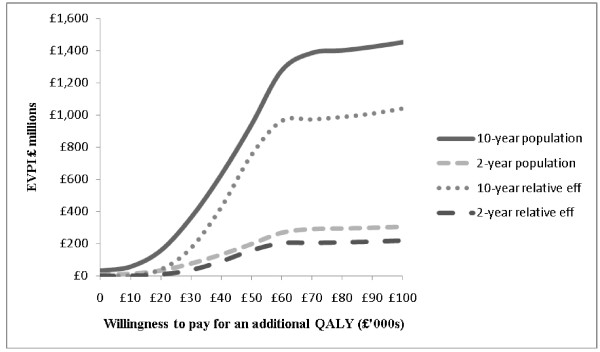
EVPI and a single EVPPI analysis based on scenario A assumptions, and an incident obese population of 308,000 people every year for 2 and 10 years, discounted at 3.5 % per annum

The per person EVPI was £11 rising to £506 at willingness’s to pay (WTP) for an additional QALY of £0 and £100,000 respectively. The population EVPI was estimated by assuming 2 and 10-year intervals, a 3.5 % annual discount rate and annual UK obesity incidence of 308,000 [37]. The corresponding population EVPI value was arguably large at all positive willingness’s to pay (Figure 4). For example the 2-year population results for the complete model was almost £33 million at a WTP of £20,000.

A single expected value of information analysis for parameters (EVPPI) was undertaken on the relative treatment effect (weighted mean difference) associated with two treatment options. The results suggest that at a willingness to pay of £20,000 to £30,000 per additional QALY, the maximum value of conducting a further RCT was between £37 M and £170 M. However, these figures reduced to between £8 M and £36 M if the time horizon was reduced to 2 years

## Discussion

The aim of this study was to assess the cost-effectiveness of e-learning devices (e-LDs) compared with conventional care (CC) in people with obesity. An economic evaluation based on discrete event simulation techniques was used to synthesise data from the published literature together with results from a systematic review and meta-analysis of RCT evidence on the impact of treatment on BMI.

The model results suggest that under most circumstances, e-LDs are unlikely to be cost-effective at any reasonable willingness-to-pay threshold. Mainly because of the fixed costs of installing the devices coupled with a negligible impact on BMI. Only when the fixed cost of the e-LD was removed, or substantially lowered, did e-learning devices appear to be cost-effective.

The fixed costs associated with the e-LDs were difficult to assess. Largely because most of the published clinical evaluations did not report resource use/cost data, they are heterogeneous in nature and it was unclear whether any were commercially available (and therefore had an associated user fee). For these reasons, the fixed cost of the e-LDs, was taken directly from the trial by McConnon - an internet-based intervention. The associated costs comprised of the development and running costs of the website over the 12 month study period. An economic evaluation was performed alongside the RCT, and it too concluded that the e-LD was unlikely to be cost-effective largely because of the fixed cost. However, it is unclear whether all e-LDs have an associated fixed cost. Second, assuming they do, fixed costs are likely to decline as the number of users increases meaning they could be substantially lower than the mean value assumed in this analysis. Third, even if the fixed cost was zero, it is unclear whether commercial programme developers would charge a fee for using the programme, how much it would be and how frequently it would be charged.

The choice of comparator programme(s) is an important design decision for any economic evaluation. Here the choice was ‘conventional care’ as delivered by health care professionals, as the control arms in the systematic review tended to use a mix of interventions. For example, most of the trials that focussed on dietary change were not particularly explicit as to what advice was given (eg. choice of diet) or who provided it (eg. a physician or nurse). This is important because it is possible that different approaches may be more or less cost-effective compared with each other, and (implicitly) averaging them as is the case here, could be misleading from an incremental perspective. Of equal importance, CC, however delivered, is not the only method of reducing weight. For example, it is possible to promote exercise, to use drug treatments such as orlistat or to use combinations of these approaches. Although the analysis containing orlistat was only crude in so much that it was not based on formal indirect treatment comparisons, it does illustrate the point that even if e-LDs were considered cost-effective compared with CC, they might not be compared with other treatments.

A number of economic evaluations of web-based interventions to promote weight loss have previously been published [24,45-47]. McConnon et al. [24] concluded that it cost about £40,000 per additional QALY if an internet programme replaced usual care, while the two other studies reported ICERs nearer US$5,000 to $7,000 per life-year gained. While these results are different to ours, there are a number of possible explanations. First, our study focused purely on e-learning devices. The other studies were not so restrictive in terms of the intervention specifics. Indeed the interventions evaluated by Krukowski [46] and Hersey [47] included an element of interaction with other patients, individual/group coaching sessions and telephone support, they were not e-learning devices. Second, our estimate of intervention effect was based on the results from a systematic review of 43 RCTs whereas the studies by McConnon and Krukowski were based on the results from single trials. Third, the study by Krukowski assumed in the base case that weight loss could not be regained, an assumption we consider to be unrealistic. Lastly, our study assessed the cost-effectiveness in people who were already considered to be obese. This is was an explicit criterion in the studies by McConnon and Hersey, but not in the evaluation by Krukowski. Indeed, the latter was said to have been performed in individuals who were highly educated, but few other details are provided. Therefore, while we have concluded that in our opinion e-LDs to promote dietary change and weight loss are unlikely to be cost-effective in obese populations, this conclusion should not necessarily be generalised to all web-based weight management interventions or to all population groups

There are undoubtedly a number of other limitations with the evidence used in the model. First, the QRISK2 risk equation is designed to assess the probability of developing primary CVD-events. Thus, the model takes does not take into account the possibility that individuals who survive one CVD event are more likely to experience another. However, it is unlikely that this would have a major bearing on the results given the negligible treatment effects.

The CVD and T2D risk equations take into account risk factors such as systolic blood pressure and cholesterol levels in addition to BMI. While the trials rarely reported changes in these risk factors, a more sophisticated modelling approach could take into account their likely correlations.

Potentially counter intuitive results were produced in a number of scenarios. For example, when the costs of T2D and CVD were increased, the ICER associated with e-learning also increased. This is because people treated with e-learning devices live longer on average with these conditions, even though they are less likely to develop them in the first instance. The net result is an increase in the incremental cost and the associated ICER. Such seemingly counter intuitive results were also reported in the NICE Obesity Guideline, along with a similar explanation [33].

The EVPI analysis suggested that the value of further research was arguably large, even with a 2 year horizon; e-based technologies are likely to have relatively short life-cycles. This is because despite the evidence of a relatively small clinical effect on BMI, the e-LDs are relatively cheap and the number of obese people is high compared with many other conditions

## Conclusion

The e-LEEM model was built to assess the cost-effectiveness of e-learning devices compared to CC for people with obesity, as methods of promoting healthier eating and weight loss. The model contains a number of assumptions and necessarily draws on evidence from a number of different sources. The results suggest that e-LDs are unlikely to be cost-effective unless they have much lower fixed costs than estimated for this analysis or future designs prove to be much more effective. Despite this, the value of further RCT-based research is high, although researchers are strongly encouraged to provide fuller descriptions of the evaluated technologies.

## Competing interests

None declared.

## Author’s contributions

AM built the economic model and drafted most sections of the report, JH, FL, EM, SM and PE designed and undertook the underpinning systematic review. EM also provided clinical expertise and knowledge about the use of e-learning devices. All authors contributed to the final write up.

## Funding source

National Institute for Health Research, Health Technology Assessment Programme

## Pre-publication history

The pre-publication history for this paper can be accessed here:

http://www.biomedcentral.com/1472-6963/12/190/prepub
